# Stochastic Model of Block Segmentation Based on Improper Quadtree and Optimal Code under the Bayes Criterion †

**DOI:** 10.3390/e24081152

**Published:** 2022-08-19

**Authors:** Yuta Nakahara, Toshiyasu Matsushima

**Affiliations:** 1 Center for Data Science, Waseda University, 1-6-1 Nisniwaseda, Shinjuku-ku, Tokyo 169-8050, Japan; 2 Department of Pure and Applied Mathematics, Waseda University, 3-4-1 Okubo, Shinjuku-ku, Tokyo 169-8555, Japan

**Keywords:** stochastic generative model, quadtree, Bayes code, lossless image compression

## Abstract

Most previous studies on lossless image compression have focused on improving preprocessing functions to reduce the redundancy of pixel values in real images. However, we assumed stochastic generative models directly on pixel values and focused on achieving the theoretical limit of the assumed models. In this study, we proposed a stochastic model based on improper quadtrees. We theoretically derive the optimal code for the proposed model under the Bayes criterion. In general, Bayes-optimal codes require an exponential order of calculation with respect to the data lengths. However, we propose an algorithm that takes a polynomial order of calculation without losing optimality by assuming a novel prior distribution.

## 1. Introduction

There are two approaches to lossless image compression. (These two approaches are detailed in Section 1 of our previous study [1].) Most previous studies (e.g., [2,3,4]) adopted an approach in which they constructed a preprocessing function $f:v^{t-1}\mapsto p$ that outputs a code length assignment vector p from past pixel values $v^{t-1}$. p determines the code length of the next pixel value $v_{t}$, or typically, a value $v_{t}^{\prime }$ equivalent to $v_{t}$ in the meaning that there exists a one-to-one mapping $\left(v_{1}^{\prime },v_{2}^{\prime },\cdots v_{t}^{\prime }\right)=g\left(v_{1},v_{2},\cdots v_{t}\right)$ computable for both encoder and decoder. Then, $v_{t}^{\prime }$ and p are passed to the following entropy coding process such as [5,6]. In this approach, the elements $p_{i}$ of the code length assignment vector p satisfy $\sum_{i}p_{i}=1$. Therefore, it appears superficially as a probability distribution. However, it does not directly govern the stochastic generation of original pixel value $v_{t}$. Hence, we cannot define the entropy of the source of pixel value $v_{t}$, and we cannot discuss the theoretical optimality of the preprocessing function $f(v^{t-1})$ and one-to-one mapping $g(v_{1},v_{2},\cdots v_{t})$.

In contrast, we adopted an approach in which we estimated a stochastic generative model $p(v_{t}|v^{t-1},\theta^{m},m)$ with an unknown parameter $\theta^{m}$ and a model variable *m*, which is directly and explicitly assumed on the original pixel value $v_{t}$ [1,7,8,9]. Therefore, we can discuss the theoretical optimality of the entire algorithm to the entropy defined from the assumed stochastic model $p(v_{t}|v^{t-1},\theta^{m},m)$. In particular, we can achieve the theoretically optimal coding under the Bayes criterion in statistical decision theory (see, e.g., [10]) by assuming prior distributions $p(\theta^{m}|m)$ and p(m) on the unknown parameter $\theta^{m}$ and model variable *m*. Such codes are known as Bayes codes [11] in information theory. It is known that the Bayes code asymptotically achieves the entropy of the true stochastic model, and its convergence speed achieves the theoretical limit [12]. The Bayes codes have shown remarkable performance in text compression (e.g., [13]). Therefore, we consider this approach.

We assume that the target image herein has non-stationarity, that is, the properties of pixel values are different among the positions in the image. For such an image, researchers have performed quadtree block segmentation as a component of preprocessing $f(v^{t-1})$ and one-to-one mapping $g(v_{1},v_{2},\cdots v_{t})$ in the former approach, and its practical efficiency has been reported in many previous studies (e.g., [4,14]). In the latter approach, we proposed a stochastic generative model $p(v_{t}|v^{t-1},\theta^{m},m)$ that contains a quadtree as a model variable *m*. By assuming a prior distribution p(m) on it, we derived the optimal code under the Bayes criterion, and we constructed a polynomial order algorithm to calculate it without loss of optimality [1]. However, in all these studies [1,4,14], the class of quadtrees is restricted to that of proper trees, whose inner nodes have exactly four children.

In this paper, we propose a stochastic generative model $p(v_{t}|v^{t-1},\theta^{m},m)$ based on an improper quadtree *m* and derive the code optimal under the Bayes criterion. In general, the codes optimal under the Bayes criterion require a summation that takes an exponential order calculation for the data length. However, we herein construct an algorithm that only requires a polynomial order calculation without losing optimality by applying a theory of probability distribution for general rooted trees [15] to the improper quadtree representing the block segmentation.

## 2. Proposed Stochastic Generative Model

Let V denote a set of possible values of a pixel. For example, we have V={0,1} for binary images and V={0,1,⋯,255} for grayscale images. Let h∈N and w∈N denote a height and a width of an image, respectively. Although our model is able to represent any rectangular images, we assume that $h=w=2^{d_{\max}}$ for $d_{\max}\in N$ in the following for the simplicity of the notation. Then, let $V_{t}$ denote the random variable of the *t*-th pixel value in order of the raster scan, and let $v_{t}\in V$ denote its realization. Note that $V_{t}$ is at the x(t)-th row and y(t)-th column, where *t* divided by *w* is x(t) with a reminder of y(t). In addition, let $V^{t}$ denote the sequence of pixel values $V_{0},V_{1},\cdots ,V_{t}$. Note that all the indices start from zero herein.

We assume $V_{t}$ is generated from a probability distribution $p(v_{t}|v^{t-1},\theta^{m},m)$ depending on an unknown model m∈M and unknown parameters $\theta^{m}\in \Theta^{m}$. (For t=0, we assume $V_{0}$ follows $p(v_{0}|\theta^{m},m)$.) We define *m* and $\theta^{m}$ in the following.

**Definition** **1** ([1]). *Let $s_{\left(x_{1}y_{1}\right)\left(x_{2}y_{2}\right)\cdots \left(x_{d}y_{d}\right)}$ denote the following index set called “block.”*

(1) $$\begin{array}{cc} s_{\left(x_{1}y_{1}\right)\left(x_{2}y_{2}\right)\cdots \left(x_{d}y_{d}\right)}:= & \left\{\left(i,j\right)\in Z^{2}\;|\;\sum_{d^{\prime }=1}^{d}\frac{x_{d^{\prime }}}{2^{d^{\prime }}}\leq \frac{i}{2^{d_{\max}}}<\left(\sum_{d^{\prime }=1}^{d}\frac{x_{d^{\prime }}}{2^{d^{\prime }}}+\frac{1}{2^{d}}\right),\right.\\ \; & \;\;\;\left.\sum_{d^{\prime }=1}^{d}\frac{y_{d^{\prime }}}{2^{d^{\prime }}}\leq \frac{j}{2^{d_{\max}}}<\left(\sum_{d^{\prime }=1}^{d}\frac{y_{d^{\prime }}}{2^{d^{\prime }}}+\frac{1}{2^{d}}\right)\right\}, \end{array}$$

*where $\left(x_{d^{\prime }}y_{d^{\prime }}\right)\in {\left\{0,1\right\}}^{2}$ for $d^{\prime }\in \left\{1,2,\cdots ,d\right\}$ and $d\leq d_{\max}$. In addition, let $s_{\lambda }$ be the set of whole indices $s_{\lambda }:=\left\{0,1,\cdots h-1\right\}\times \left\{0,1,\cdots ,w-1\right\}$. Then, let S denote the set that consists of all the above index sets, that is, $S:=\{s_{\lambda },s_{\left(00\right)},\cdots ,s_{\left(11\right)},s_{\left(00)(00\right)},\cdots ,$$s_{\left(11)(11\right)},\cdots ,s_{\left(11)(11)\cdots (11\right)}\}$.*

**Example** **1** ([1]). *For $d_{\max}=2$,*

(2) $$\begin{array}{c} s_{\left(01\right)}=\left\{\left(i,j\right)\in Z^{2}|0\leq i<2,2\leq j<4\right\}=\left\{\left(0,2\right),\left(0,3\right),\left(1,2\right),\left(1,3\right)\right\}. \end{array}$$

*Therefore, it represents the indices of the upper right region. In a similar manner, $s_{\left(01)(11\right)}=\left\{\left(i,j\right)\in Z^{2}|1\leq i<2,3\leq j<4\right\}=\left\{\left(1,3\right)\right\}$. It should be noted that the cardinality |s| for each s∈S represents the number of pixels in the block.*

**Figure 1:**
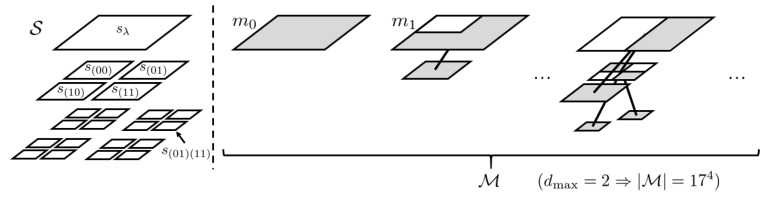
An example of node set S and models *m*. The set of blocks with gray region corresponds to $U^{m}$, which covers the whole region of the image and represents a block segmentation pattern.

**Definition** **2.** *We define the model m as a quadtree whose nodes are elements of S. Let M denote the set of the models. Let $S^{m}\subset S$, $L^{m}\subset S$ and $I^{m}\subset S$ denote the set of the nodes, the leaf nodes and the inner nodes of m∈M, respectively. Let $U^{m}\subset S^{m}$ denote the set of nodes that have less than four children. Then, $U^{m}$ corresponds to a pattern of variable block size segmentation, as shown in Figure 1.*

**Definition** **3.** *Each node $s\in U^{m}$ of the model m has a parameter $\theta_{s}^{m}$ whose parameter space is $\Theta_{s}^{m}$. We define $\theta^{m}$ as a tuple of parameters ${\left\{\theta_{s}^{m}\right\}}_{s\in U^{m}}$, and let $\Theta^{m}$ denote its space.*

Notably, we can reduce the number of parameters from an equivalent model represented by a proper tree with added dummy child nodes. See the following example.

**Figure 2:**
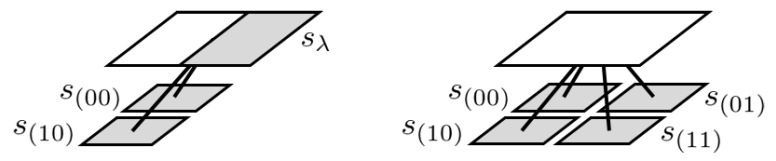
A model with three parameters (**left**) and a model with four parameters (**right**).

**Example** **2.** *For $d_{\max}=2$, consider a model represented by the left-hand side image in Figure 2. It has three parameters: $\theta_{s_{\lambda }}$, $\theta_{s_{\left(00\right)}}$, and $\theta_{s_{\left(10\right)}}$. An equivalent model can be represented by a proper quadtree shown in the right-hand side of Figure 2, if assuming $\theta_{s_{\left(01\right)}}=\theta_{s_{\left(11\right)}}$ by chance. However, it requires four parameters: $\theta_{s_{\left(00\right)}}$, $\theta_{s_{\left(01\right)}}$, $\theta_{s_{\left(10\right)}}$, and $\theta_{s_{\left(11\right)}}$. Therefore, it causes inefficient learning.*

Under the model m∈M and the parameters $\theta^{m}\in \Theta^{m}$, we assume that the *t*-th pixel value $V_{t}$ is generated as follows.

**Assumption** **A1.** *We assume that*

(3) $$\begin{array}{c} p\left(v_{t}|v^{t-1},\theta^{m},m\right)=p\left(v_{t}|v^{t-1},\theta_{s}^{m}\right), \end{array}$$

*where s is the minimal block that satisfies $\left(x\left(t\right),y\left(t\right)\right)\in s\in U^{m}$ (in other words, s is the the deepest node that contains (x(t),y(t)) in m). For t=0, we assume a similar condition $p\left(v_{0}|\theta^{m},m\right)=p\left(v_{0}|\theta_{s}^{m}\right)$.*

Thus, the pixel value $V_{t}$ given the past sequence $V^{t-1}$ depends only on the parameter of the minimal block *s* that contains $V_{t}$. Note that we do not assume a specific form of $p(v_{t}|v^{t-1},\theta_{s}^{m})$ at this point. For example, we can assume the Bernoulli distribution for V={0,1} and also the Gaussian distribution (with an appropriate normalization and quantization) for V={0,1,⋯,255}.

## 3. The Bayes Code for Proposed Model

Since the true *m* and $\theta^{m}$ are unknown, we assume prior distributions p(m) and $p(\theta^{m}|m)$. Then, we estimate the true generative probability $p(v_{t}|v^{t-1},\theta^{m},m)$ by $q(v_{t}|v^{t-1})$ under the Bayes criterion in statistical decision theory (see, e.g., [10]). Subsequently, we use $q(v_{t}|v^{t-1})$ as a coding probability of the entropy code such as [16]. Such a code is known as Bayes codes [11] in information theory. The expected code length of the Bayes code converges to the entropy of $p(v_{t}|v^{t-1},\theta^{m},m)$ for sufficiently large data length, and its convergence speed achieves the theoretical limit [12]. The Bayes code has shown remarkable performances in text compression (e.g., [13]).

The optimal coding probability of the Bayes code for $v_{t}$ is derived as follows, according to the general formula in [11].

**Proposition** **1.** The optimal coding probability $q^{*}\left(v_{t}|v^{t-1}\right)$ under the Bayes criterion is given by

(4) $$\begin{array}{c} q^{*}\left(v_{t}|v^{t-1}\right)=\sum_{m\in M}p\left(m|v^{t-1}\right)\int p\left(v_{t}|v^{t-1},\theta^{m},m\right)p\left(\theta^{m}|v^{t-1},m\right)d\theta^{m}. \end{array}$$

We call $q^{*}\left(v_{t}|v^{t-1}\right)$ the Bayes-optimal coding probability.

Proposition 1 implies that we should use the coding probability that is a weighted mixture of $p(v_{t}|v^{t-1},\theta^{m},m)$ for every block segmentation pattern *m* and parameters $\theta^{m}$ according to the posteriors $p(m|v^{t-1})$ and $p(\theta^{m}|v^{t-1},m)$. (For t=0, $p(v_{0}|\theta^{m},m)$ is mixed with weights according to the priors p(m) and $p(\theta^{m}|m)$, which corresponds to the initialization of the algorithm.) Notably, M is generalized to the set of improper quadtrees from the set of proper quadtrees although (4) has a similar form to Formula (5) in [1].

## 4. Polynomial Order Algorithm to Calculate Bayes-Optimal Coding Probability

Unfortunately, the Bayes-optimal coding probability (4) contains a computationally hard calculation. (Herein, we assume that $\int p\left(v_{t}|v^{t-1},\theta^{m},m\right)p\left(\theta^{m}|v^{t-1},m\right)d\theta^{m}$ is feasible. Examples of feasible settings will be described in the next section.) The summation cost for *m* exponentially increases with respect to $d_{\max}$. Therefore, we propose a polynomial order algorithm to calculate (4) without loss of optimality by applying a theory of probability distribution for general rooted trees [15] to the improper quadtree *m*. In this section, we focus on the procedure of the constructed algorithm. Its validity is described in Appendix A.

**Figure 3:**
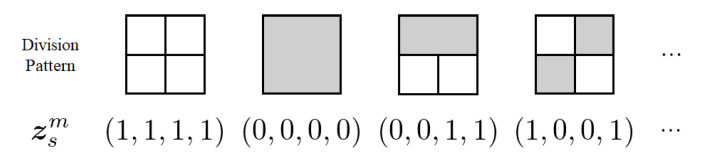
Examples of block division patterns and corresponding $z_{s}^{m}$.

**Definition** **4.** *Let $\mathrm{Ch}\left(s\right):=\left\{s_{\left(00\right)},s_{\left(01\right)},s_{\left(10\right)},s_{\left(11\right)}\right\}$ be the set of child nodes of s. We define a vector $z_{s}^{m}\in {\left\{0,1\right\}}^{4}$ representing the block division pattern of s in $S^{m}$ as $z_{s}^{m}:={\left(z_{ss^{\prime }}^{m}\right)}_{s^{\prime }\in \mathrm{Ch}\left(s\right)}:=\left(I\left\{s_{\left(00\right)}\in S^{m}\right\},I\left\{s_{\left(01\right)}\in S^{m}\right\},I\left\{s_{\left(10\right)}\in S^{m}\right\},I\left\{s_{\left(11\right)}\in S^{m}\right\}\right)$, where I{·} denotes the indicator function. Examples of $z_{s}^{m}$ are shown in Figure 3. For leaf nodes, $z_{s}^{m}=0$.*

First, we assume the following prior distributions as p(m) and $p(\theta^{m}|m)$.

**Assumption** **A2.** *Let $\eta_{s}\left(z\right)\in \left[0,1\right]$ be a given hyper parameter of a block s∈S, which satisfies $\sum_{z\in {\left\{0,1\right\}}^{4}}\eta_{s}\left(z\right)=1$. Then, we assume that the prior on M is represented as follows.*

(5) $$\begin{array}{c} p\left(m\right)=\prod_{s\in S_{m}}\eta_{s}\left(z_{s}^{m}\right), \end{array}$$

*where $\eta_{s}\left(0\right)=1$ for s whose cardinality |s| is equal to 1.*

Intuitively, $\eta_{s}\left(z_{s}^{m}\right)$ represents the conditional probability that *s* has the block division pattern $z_{s}^{m}$ under the condition that $s\in S^{m}$. The above prior actually satisfies the condition $\sum_{m\in M}p\left(m\right)=1$. Although this is proved for any rooted tree in [15], we briefly describe a proof restricted for our model in the Appendix A to make this paper self-contained. Note that the above assumption does not restrict the expressive capability of the general prior in the meaning that each model *m* still has possibly to be assigned a non-zero probability p(m)>0.

**Assumption** **A3.** *For each model m∈M, we assume that*

(6) $$\begin{array}{c} p\left(\theta^{m}|m\right)=\prod_{s\in U^{m}}p\left(\theta_{s}^{m}|m\right). \end{array}$$

*Moreover, for any $m,m^{\prime }\in M$, $s\in U^{m}\cap U^{m^{\prime }}$, and $\theta_{s}\in \Theta_{s}$, we assume that*

(7) $$\begin{array}{c} p\left(\theta_{s}|m\right)=p\left(\theta_{s}|m^{\prime }\right)=:p_{s}\left(\theta_{s}\right). \end{array}$$

Therefore, each element $\theta_{s}^{m}$ of the parameters $\theta^{m}$ depends only on *s* and they are independent from both of the other elements and the model *m*.

From Assumptions 1 and 3, the following lemma holds.

**Lemma** **1.** *For any $m,m^{\prime }\in M$, let $s_{t}\in U^{m}$ and $s_{t}^{\prime }\in U^{m^{\prime }}$ denote the minimal node that satisfies $\left(x\left(t\right),y\left(t\right)\right)\in s_{t}\in U^{m}$ and $\left(x\left(t\right),y\left(t\right)\right)\in s_{t}^{\prime }\in U^{m^{\prime }}$, respectively. If $s_{t}=s_{t}^{\prime }=:s$ and $z_{s_{t}}^{m}=z_{s_{t}^{\prime }}^{m^{\prime }}=:z_{s}$, that is, they are the same block and their division patterns are also the same, then*

(8) $$p(v_{t}|v^{t-1},m)=p(v_{t}|v^{t-1},m^{\prime }).$$

*Hence, we represent it by $\tilde{q}\left(v_{t}|v^{t-1},s,z_{s}\right)$ because it does not depend on m but $\left(s,z_{s}\right)$. Let $\tilde{q}\left(v_{t}|v^{t-1},s\right)=p\left(v_{t}|m\right)=p\left(v_{t}|m^{\prime }\right)$ for t=0.*

Lemma 1 means that the optimal coding probability for $v_{t}$ depends on the minimal block *s* that contains $v_{t}$ and its division pattern $z_{s}$. Therefore, it could be calculated as $\tilde{q}\left(v_{t}|v^{t-1},s,z_{s}\right)$ if $\left(s,z_{s}\right)$ was known.

At last, the Bayes-optimal coding probability $q^{*}\left(v_{t}|v^{t-1}\right)$ can be calculated by a recursive function for nodes on a path of the perfect quadtree on S. The definition of the path is the same as [1].

**Figure 4:**
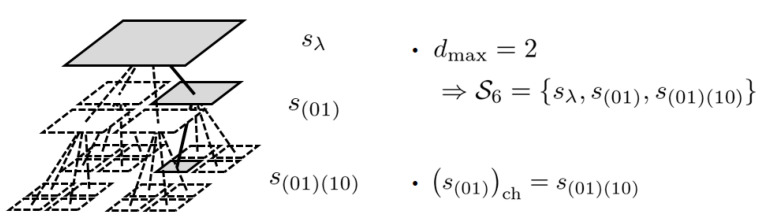
An example of a path constructed from $S_{t}$.

**Definition** **5** ([1]). *Let $S_{t}$ denote the set of nodes which contain (x(t),y(t)). They construct a path from the leaf node $s_{\left(x_{1}y_{1}\right)\left(x_{2}y_{2}\right)\cdots \left(x_{d_{\max}}y_{d_{\max}}\right)}=\left\{\left(x\left(t\right),y\left(t\right)\right)\right\}$ to the root node $s_{\lambda }$ on the perfect quadtree whose depth is $d_{\max}$ on S, as shown in Figure 4. In addition, let $s_{\mathrm{ch}}\in S_{t}$ denote the child node of $s\in S_{t}$ on that path.*

**Definition** **6.** *We define the following recursive function $q(v_{t}|v^{t-1},s)$ for $s\in S_{t}$.*

(9) $$\begin{array}{c} q\left(v_{t}|v^{t-1},s\right):=\left\{\begin{array}{cc} \tilde{q}\left(v_{t}|v^{t-1},s,0\right), & |s|=1,\\ \sum_{z_{s}:z_{ss_{\mathrm{ch}}}=0}\eta_{s}\left(z_{s}|v^{t-1}\right)\tilde{q}\left(v_{t}|v^{t-1},s,z_{s}\right)\\ \;+\left(\sum_{z_{s}:z_{ss_{\mathrm{ch}}}=1}\eta_{s}\left(z_{s}|v^{t-1}\right)\right)q\left(v_{t}|v^{t-1},s_{\mathrm{ch}}\right), & \mathrm{otherwise}, \end{array}\right. \end{array}$$

*where $\eta_{s}\left(z_{s}|v^{t}\right)$ is also recursively updated for $s\in S_{t}$ as follows:*

(10) $$\begin{array}{c} \eta_{s}\left(z_{s}|v^{t}\right):=\left\{\begin{array}{cc} \eta_{s}\left(z_{s}\right), & t=-1,\\ \frac{\eta_{s}\left(z_{s}|v^{t-1}\right)\tilde{q}\left(v_{t}|v^{t-1},s,z_{s}\right)}{q(v_{t}|v^{t-1},s)}, & t\geq 0\wedge z_{ss_{\mathrm{ch}}}=0,\\ \frac{\eta_{s}\left(z_{s}|v^{t-1}\right)q\left(v_{t}|v^{t-1},s_{\mathrm{ch}}\right)}{q(v_{t}|v^{t-1},s)}, & t\geq 0\wedge z_{ss_{\mathrm{ch}}}=1. \end{array}\right. \end{array}$$

Consequently, the following theorem holds.

**Theorem** **1.** The Bayes-optimal coding probability $q^{*}\left(v_{t}|v^{t-1}\right)$ for the proposed model is calculated by

(11) $$\begin{array}{c} q^{*}\left(v_{t}|v^{t-1}\right)=q\left(v_{t}|v^{t-1},s_{\lambda }\right). \end{array}$$

Although Theorem 1 is proved by applying Corollary 2 of Theorem 7 in [15], we briefly describe a proof restricted to our model in the Appendix A to make this paper self-contained. Theorem 1 means that the summation with respect to m∈M in (4) is able to be replaced by the summation with respect to $s\in S_{t}$ and $z_{s}\in {\left\{0,1\right\}}^{4}$, which costs only $O(2^{4}d_{\max})$. The proposed algorithm recursively calculates a weighted mixture of coding probabilities $\tilde{q}\left(v_{t}|v^{t-1},s,z_{s}\right)$ for the case where block *s* is not divided at $s_{\mathrm{ch}}$ (i.e., $z_{ss_{\mathrm{ch}}}=0$) and the coding probability $q(v_{t}|v^{t-1},s_{\mathrm{ch}})$ for the case where block *s* is divided at $s_{\mathrm{ch}}$ (i.e., $z_{ss_{\mathrm{ch}}}=1$).

## 5. Experiments

In this section, we perform four experiments. Three of them are similar to the experiments in [1]. The fourth one is newly added. In Experiments 1, 2, and 3, we assume V={0,1}, which is the simplest setting, to focus on the effect of the improper quadtrees. In Experiment 4, we assume V={0,1,⋯,255} to show our method is also applicable to grayscale images. The purpose of the first experiment is to confirm the Bayes optimality of $q(v_{t}|v^{t-1},s_{\lambda })$ for synthetic images generated from the proposed model. The purpose of the second experiment is to show an example image suitable to our model. The purpose of the third experiment is to compare average coding rates of our proposed algorithm with a current image coding procedure on real images. The purpose of the fourth experiment is to show our method is applicable to grayscale images.

In Experiments 1 and 2, $p(v_{t}|v^{t-1},\theta^{m},m)$ is Bernoulli distribution $\mathrm{Bern}(v_{t}|\theta_{s}^{m})$ for the minimal *s* that satisfies $\left(x\left(t\right),y\left(t\right)\right)\in s\in U^{m}$. Each element of $\theta^{m}$ is i.i.d. distributed with the beta distribution Beta(θ|α,β), which is the conjugate prior distribution of Bernoulli distribution. Therefore, the integral in (4) has a closed form. The hyperparameter $\eta_{s}\left(z\right)$ of the model prior is $\eta_{s}\left(z\right)=1/2^{4}$ for every s∈S and $z\in {\left\{0,1\right\}}^{4}$, and the hyperparameters of the beta distribution are α=β=1/2. For comparison, we used the previous method based on proper quadtrees, whose hyperparameters are the same as the experiments in [1], and the standard methods known as JBIG [17] and JBIG2 [18].

### 5.1. Experiment 1

The setting of Experiment 1 is as follows. The width and height of images are w=h=$2^{d_{\max}}=64$. We generate 1000 images according to the following procedure.

1. Generate *m* according to (5).
2. Generate $\theta_{s}^{m}$ according to $p(\theta_{s}^{m}|m)$ for $s\in U^{m}$.
3. Generate pixel value $v_{t}$ according to $p(v_{t}|v^{t-1},\theta^{m},m)$ for t∈{0,1,⋯,hw−1}.
4. Repeat Steps 1 to 3 for 1000 times.

**Figure 5:**
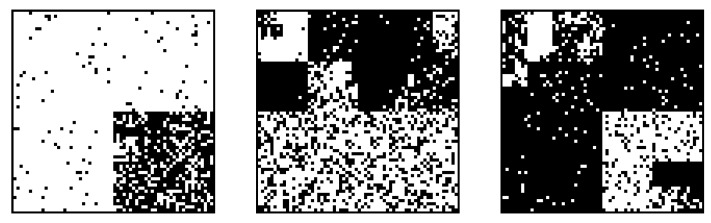
Examples of the generated images in Experiment 1.

Examples of the generated images are shown in Figure 5. Subsequently, we compress these 1000 images. The size of the image is saved in the header of the compressed file using 4 bytes. The coding probability calculated by the proposed algorithm is quantized in $2^{16}$ levels and substituted into the range coder [16]. Table 1 shows the coding rates (bit/pel) averaged over all the images. Our proposed code has the minimum coding rate as expected by the Bayes optimality.

### 5.2. Experiment 2

**Figure 6:**
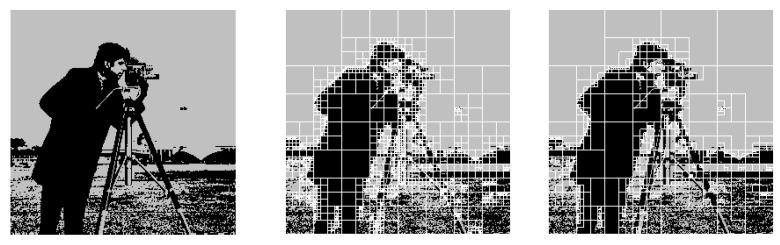
The original image (**left**), the MAP estimated model $m^{\mathrm{MAP}}$ based on the proper quadtree [1] (**middle**), and that based on the improper quadtree (**right**).

In Experiment 2, we compress camera.tif in [19], which is binarized with the threshold of 128. The setting of the header and the range coder is the same as those of Experiment 1. Figure 6 visualizes the maximum a posteriori (MAP) estimation $m^{\mathrm{MAP}}=\arg\max_{m}p\left(m|v^{hw-1}\right)$ based on the improper quadtree model and the proper quadtree model [1], which are by-products of the compression. They are obtained by applying Theorem 3 in [15] and the algorithm in Appendix B in the preprint of the full version of [15], which is uploaded on arXiv. The improper quadtree represents the non-stationarity by a fewer number of regions (i.e., fewer parameters) than that of the proper quadtree [1]. Table 2 shows that the coding rate of our proposed model for camera.tif is lower than the previous one based on the proper quadtree [1] and JBIG [17] without any special tuning. However, JBIG2 [18] showed the lowest coding rate. The improvement of our method for real images will be described in the next experiment.

### 5.3. Experiment 3

In Experiment 3, we compare the proposed algorithm with the proper-quadtree-based algorithm [1], JBIG [17], and JBIG2 [18] on real images from [19]. They are binarized in a similar manner to Experiment 2. The setting of the header and the range coder is the same as those of Experiments 1 and 2. A difference from Experiments 1 and 2 is in the stochastic generative model $p(v_{t}|v^{t-1},\theta^{m},m)$ assumed on each block *s*. We assume another model $p(v_{t}|v^{t-1},\theta^{m},m)$ represented as the Bernoulli distribution $\mathrm{Bern}(v_{t}|\theta_{s;v_{t-w-1}v_{t-w}v_{t-w+1}v_{t-1}}^{m})$ that depends on the neighboring four pixels. (If the indices go out of the image, we use the nearest past pixel in Manhattan distance.) Therefore, $p(v_{t}|v^{t-1},\theta^{m},m)$ has a kind of Markov property. In other words, there are 16 parameters $\theta_{s;0000}^{m},\theta_{s;0001}^{m},\cdots ,\theta_{s;1111}^{m}$ for each block *s* of model *m*, and one of them is chosen by the observed values $v_{t-w-1}$, $v_{t-w}$, $v_{t-w+1}$, and $v_{t-1}$ in the past. Each parameter is i.i.d. distributed with the beta distribution whose parameters are α=β=1/2. The results are shown in Table 3. The algorithms labeled as Improper-i.i.d. and Proper-i.i.d. are the same as those in Experiments 1 and 2. The algorithms labeled as Improper-Markov and Proper-Markov are the aforementioned ones.

Improper-Markov outperforms the other methods from the perspective of average coding rates. The effect of the improper quadtree is probably amplified because the number of parameters for each block is increased. However, JBIG2 [18] still outperforms our algorithms only for text. We consider it is because JBIG2 [18] is designed for text images such as faxes in contrast to our general-purpose algorithm. Note that our algorithm has room for improvement by tuning the hyperparameters α and β of the beta distribution for each of $\theta_{s;0000}^{m},\theta_{s;0001}^{m},\cdots ,\theta_{s;1111}^{m}$.

### 5.4. Experiment 4

Through Experiment 4, we show our method is applicable to grayscale images. Herein, we assume two types of stochastic generative models $p(v_{t}|v^{t-1},\theta^{m},m)$ for the block of the proper quadtree and the improper quadtree. The first one is the i.i.d. Gaussian distribution $N(v_{t}|\mu_{s}^{m},{\left(\lambda_{s}^{m}\right)}^{-1})$. In this case, $\theta_{s}^{m}$ can be regarded as $\left\{\mu_{s}^{m},\lambda_{s}^{m}\right\}\in R\times R_{>0}$. The second one is the two-dimensional autoregressive (AR) model [7] of the neighboring four pixels, i.e., $N(v_{t}|{\tilde{v}}_{t-1}^{⊤}w_{s}^{m},{\left(\tau_{s}^{m}\right)}^{-1})$, where ${\tilde{v}}_{t-1}={\left(v_{t-w-1},v_{t-w},v_{t-w+1},v_{t-1}\right)}^{⊤}$. (If the indices go out of the image, we use the nearest past pixel in Manhattan distance.) In this case, $\theta_{s}^{m}$ can be regarded as $\left\{w_{s}^{m},\tau_{s}^{m}\right\}\in R^{4}\times R_{>0}$. For both models, $v_{t}$ is normalized and quantized into V={0,1,⋯,255} in a similar manner to [7]. The prior distributions for each model are assumed to be the Gauss–gamma distributions $N\left(\mu_{s}^{m}|\mu_{0},{\left(\kappa_{0}\lambda_{s}\right)}^{-1}\right)\mathrm{Gam}\left(\lambda_{s}^{m}|\alpha_{0},\beta_{0}\right)$ and $N\left(w_{s}^{m}|\mu_{0},{\left(\tau_{s}^{m}\Lambda_{0}\right)}^{-1}\right)\mathrm{Gam}\left(\tau_{s}^{m}|\alpha_{0},\beta_{0}\right)$, where $\mu_{0}=0$, $\mu_{0}=0$, $\kappa_{0}=0.01$, $\Lambda_{0}=0.01I$, $\alpha_{0}=1.0$, $\beta_{0}=0.0001$. Here, I is the identity matrix. The results are shown in Table 4. (The values for previous studies [2,4,20,21] are cited from [21].)

The coding rates of the proper-quadtree-based algorithm are improved by our proposed method for all the images in this data set and for both settings of the stochastic generative model assumed within blocks. This indicates the superiority of the improper-quadtree-based model to the proper-quadtree-based model. The method labeled by Improper-AR showed an average coding rate lower than JPEG2000, averaging for the whole images. It also showed an average coding rate lower than JPEG-LS, averaging for the natural images. Although it does not outperform recent methods such as MRP and Vanilc, we consider this is because of the suitability of the stochastic generative model within blocks, which is out of the scope of this paper.

## 6. Conclusions

We proposed a novel stochastic model based on the improper quadtree, so that our model effectively represents the variable block size segmentation of images. Then, we constructed a Bayes code for the proposed stochastic model. Moreover, we introduced an algorithm to implement it in polynomial order of data size without loss of optimality. Some experiments both on synthetic and real images demonstrated the flexibility of our stochastic model and the efficiency of our algorithm. As a result, the derived algorithm showed a better average coding rate than that of JBIG2 [18].

## Figures and Tables

**Table 1:** The average coding rates (bit/pel).

Improper Quadtree (Proposal)	Proper Quadtree [1]	JBIG [17]	JBIG2 [18]
**0.619**	0.624	1.811	0.962

**Table 2:** The coding rates for the camera.tif in [19] (bit/pel).

Improper Quadtree (Proposal)	Proper Quadtree [1]	JBIG [17]	JBIG2 [18]
0.318	0.323	0.348	**0.293**

**Table 3:** The coding rates for the binarized images from [19] (bit/pel).

Images	Proper-i.i.d	Improper-i.i.d.	JBIG [17]	Proper-Markov	JBIG2 [18]	Improper-Markov
bird	0.121	0.113	0.149	0.099	0.090	**0.067**
bridge	0.390	0.382	0.386	0.373	0.353	**0.300**
camera	0.323	0.318	0.348	0.310	0.293	**0.255**
circles	0.100	0.090	0.102	0.060	0.045	**0.030**
crosses	0.140	0.132	0.083	0.110	0.027	**0.027**
goldhill1	0.371	0.364	0.359	0.353	0.321	**0.280**
horiz	0.075	0.070	0.078	0.022	0.018	**0.004**
lena1	0.254	0.243	0.217	0.216	0.169	**0.141**
montage	0.176	0.165	0.164	0.163	0.114	**0.087**
slope	0.091	0.083	0.096	0.056	0.038	**0.021**
squares	0.005	0.004	0.076	0.010	0.016	**0.003**
text	0.468	0.465	0.301	0.468	**0.229**	0.280
avg.	0.209	0.202	0.197	0.187	0.143	**0.125**

**Table 4:** The coding rates for the grayscale images from [19] (bit/pel).

Images	JPEG2000 [20]	JPEG-LS [2]	MRP [4]	Vanilc [21]	Proper-Gaussian	Improper-Gaussian	Proper-AR	Improper-AR
bird	3.630	3.471	3.238	**2.749**	4.086	4.055	3.461	3.422
bridge	6.012	5.790	**5.584**	5.596	6.353	6.294	5.696	5.678
camera	4.570	4.314	3.998	**3.995**	4.651	4.589	4.163	4.121
circles	0.928	0.153	0.132	**0.043**	1.190	0.915	1.030	0.826
crosses	1.066	0.386	0.051	**0.016**	1.603	1.240	0.898	0.625
goldhill1	5.516	5.281	5.098	**5.090**	5.796	5.738	5.220	5.196
horiz	0.231	0.094	0.016	**0.015**	1.091	0.922	0.279	0.216
lena1	4.755	4.581	4.189	**4.123**	5.312	5.259	4.433	4.394
montage	2.983	2.723	**2.353**	2.363	3.818	3.734	2.940	2.850
slope	1.342	1.571	**0.859**	0.960	3.721	3.683	1.728	1.602
squares	0.163	0.077	0.013	**0.007**	0.335	0.205	0.323	0.202
text	4.215	1.632	3.175	**0.621**	4.310	3.691	4.176	3.732
Whole avg.	2.951	2.506	2.392	**2.132**	3.522	3.360	2.862	2.739
Natural avg.	4.897	4.687	4.421	**4.311**	5.240	5.187	4.595	4.562
Artificial avg.	1.561	0.948	0.943	**0.575**	2.295	2.056	1.625	1.436

## Data Availability

Publicly available datasets were analyzed in this study. This data can be found here: http://links.uwaterloo.ca/Repository.html (accessed on 18 August 2022).

## Appendix A. Validity of Proposed Algorithm

### Validity of Prior Distribution for Models

Although a general proof for any rooted trees is described in [15] (please see also a preprint for the full version of [15] uploaded on arXiv.), in the following, we briefly describe a proof restricted for our model to make this paper self-contained.

(A1) $$\begin{array}{cc} \sum_{m\in M}p\left(m\right)=\underset{\left(a\right)}{\underset{︸}{\sum_{m\in M}\prod_{s\in S^{m}}\eta_{s}\left(z_{s}^{m}\right)}} & =\sum_{z_{s_{\lambda }}\in {\left\{0,1\right\}}^{4}}\sum_{m\in M:z_{s_{\lambda }}^{m}=z_{s_{\lambda }}}\prod_{s\in S^{m}}\eta_{s}\left(z_{s}^{m}\right) \end{array}$$

(A2) $$\begin{array}{cc} \; & =\sum_{z_{s_{\lambda }}\in {\left\{0,1\right\}}^{4}}\eta_{s_{\lambda }}\left(z_{s_{\lambda }}\right)\sum_{m\in M:z_{s_{\lambda }}^{m}=z_{s_{\lambda }}}\prod_{s\in S^{m}\setminus \left\{s_{\lambda }\right\}}\eta_{s}\left(z_{s}^{m}\right) \end{array}$$

(A3) $$\begin{array}{cc} \; & =\sum_{z_{s_{\lambda }}\in {\left\{0,1\right\}}^{4}}\eta_{s_{\lambda }}\left(z_{s_{\lambda }}\right)\prod_{s^{\prime }\in \mathrm{Ch}\left(s_{\lambda }\right)}{\left(\underset{\left(b\right)}{\underset{︸}{\sum_{m\in M^{s^{\prime }}}\prod_{s\in S^{m}}\eta_{s}\left(z_{s}^{m}\right)}}\right)}^{z_{s_{\lambda }s^{\prime }}}\; \end{array}$$

In (A3), $M^{s^{\prime }}$ denotes the set of subtrees whose root node is $s^{\prime }$. The factorization from (A2) to (A3) is because *m* in (A2) is determined by the subtrees $m^{\prime }$ whose root nodes are in $\mathrm{Ch}(s_{\lambda })$. The same idea is also detailed in Figure 4 in the preprint of the full version of [15], which is uploaded on arXiv. The underbraced parts (a) and (b) have the same structure except for the depth of the root node. We represent them by ϕ(s), which is a function of the root node *s* of the subtree.

Subsequently, we have

(A4) $$\begin{array}{c} \phi \left(s\right)=\left\{\begin{array}{cc} \sum_{z\in {\left\{0,1\right\}}^{4}}\eta_{s}\left(z\right)=1, & |s|=1,\\ \sum_{z\in {\left\{0,1\right\}}^{4}}\eta_{s}\left(z\right)\prod_{s^{\prime }\in \mathrm{Ch}\left(s\right)}{\left(\phi (s^{\prime })\right)}^{z_{ss^{\prime }}}, & \mathrm{otherwise}. \end{array}\right. \end{array}$$

Therefore, the following holds by recursively substituting ϕ(s) from the leaf nodes.

(A5) $$\begin{array}{c} \sum_{m\in M}p\left(m\right)=\phi \left(s_{\lambda }\right)=1 \end{array}$$

**Proof** **of** **Lemma** **1.** Let $R(s,z_{s})$ denote ${⋃}_{s^{\prime }\in \mathrm{Ch}\left(s\right):z_{ss^{\prime }}=0}s^{\prime }$, which is a region where $v_{t}$ is generated according to $\eta_{s}\left(z_{s}\right)$ when (x(t),y(t))∈s. Then,

(A6) $$\begin{array}{cc} p(v_{t}|v^{t-1},m) & \propto \int p\left(v_{t}|v^{t-1},\theta_{s}^{m}\right)\int p\left(\theta^{m}|m\right)p\left(v^{t-1}|\theta^{m},m\right)d\theta_{\setminus s}^{m}d\theta_{s}^{m} \end{array}$$

(A7) $$\begin{array}{cc} \; & \propto \int p\left(v_{t}|v^{t-1},\theta_{s}^{m}\right)p_{s}\left(\theta_{s}^{m}\right)\prod_{i\in \{i^{\prime }\leq t|\left(x\left(i^{\prime }\right),y\left(i^{\prime }\right)\right)\in R\left(s,z_{s}\right)\}}p\left(v_{i}|v^{i-1},\theta_{s}^{m}\right)d\theta_{s}^{m}, \end{array}$$

where ∝ means that the left-hand side is proportional to the right-hand side, regarding the variables except for $v_{t}$ as constant, and $\theta_{\setminus s}^{m}$ denotes the parameters $\theta^{m}$ except for $\theta_{s}^{m}$. Formula (A7) does not depend on *m* but $\left(s,z_{s}\right)$. □

**Proof** **of** **Theorem** **1.** Although Theorem 1 is proved by applying Corollary 2 of Theorem 7 in [15] (please see also the preprint for the full version of [15] uploaded on arXiv), in the following, we briefly describe a proof restricted to our model to make this paper self-contained. Theorem 1 will be proved by induction. First, we assume

(A8) $$\begin{array}{c} p\left(m|v^{t-1}\right)=\prod_{s\in S^{m}}\eta_{s}\left(z_{s}^{m}|v^{t-1}\right), \end{array}$$

which is true for t=0 because of Assumption 2 and will be proved later for t>0. In addition, we define the following function to simplify the notation.

(A9) $$\begin{array}{c} f\left(v_{t}|v^{t-1},s,z_{s}\right):=\left\{\begin{array}{cc} \tilde{q}\left(v_{t}|v^{t-1},s,0\right), & s=\{(x(t),y(t))\},\\ \tilde{q}\left(v_{t}|v^{t-1},s,z_{s}\right), & {}^{\exists }s^{\prime }\in \mathrm{Ch}\left(s\right)\;s.t.\;\left(s^{\prime }∋\left(x\left(t\right),y\left(t\right)\right)\right)\wedge \left(z_{ss^{\prime }}=0\right),\\ 1, & \mathrm{otherwise}. \end{array}\right. \end{array}$$

Using this notation, we can represent $p(v_{t}|v^{t-1},m)$ as follows.

(A10) $$\begin{array}{c} p\left(v_{t}|v^{t-1},m\right)=\prod_{s\in S^{m}}f\left(v_{t}|v^{t-1},s,z_{s}^{m}\right). \end{array}$$

(Equations (A9) and (A10) correspond to Conditions 4 and 3 in [15], respectively. If we accept this fact, Theorem 1 is immediately proved by applying Corollary 2 in [15].) By using (A10), we have

(A11) $$\begin{array}{cc} p(v_{t}|v^{t-1}) & =\sum_{m\in M}p\left(m|v^{t-1}\right)p\left(v_{t}|v^{t-1},m\right)=\sum_{m\in M}\prod_{s\in S^{m}}\eta_{s}\left(z_{s}^{m}|v^{t-1}\right)f\left(v_{t}|v^{t-1},s,z_{s}^{m}\right). \end{array}$$

Since the right-hand side of (A11) has a similar form to the underbraced part (a) in (A2), we can define a recursive function $q(v_{t}|v^{t-1},s)$ that satisfies

(A12) $$\begin{array}{c} p\left(v_{t}|v^{t-1}\right)=q\left(v_{t}|v^{t-1},s_{\lambda }\right), \end{array}$$

where

(A13) $$\begin{array}{c} q\left(v_{t}|v^{t-1},s\right):=\left\{\begin{array}{cc} f(v_{t}|v^{t-1},s,0), & |s|=1\\ \sum_{z_{s}\in {\left\{0,1\right\}}^{4}}\eta_{s}\left(z_{s}|v^{t-1}\right)f\left(v_{t}|v^{t-1},s,z_{s}\right)\\ \;\;\;\times \prod_{s^{\prime }\in \mathrm{Ch}\left(s\right)}q{\left(v_{t}|v^{t-1},s^{\prime }\right)}^{z_{ss^{\prime }}}, & \mathrm{otherwise} \end{array}\right. \end{array}$$

By substituting (A9), $q(v_{t}|v^{t-1},s)=1$ holds for s∌(x(t),y(t)) (or equivalently for $s\notin S_{t}$). Therefore, we need not calculate (A13) for $s\notin S_{t}$ and (9) will be derived by substituting (A9) again for $s\in S_{t}$. Lastly, we will prove (A8). Using (A9), the updating Formula (10) can be generally represented as follows.

(A14) $$\begin{array}{c} \eta_{s}\left(z_{s}|v^{t}\right)=\left\{\begin{array}{cc} \eta_{s}\left(z_{s}\right), & t=-1,\\ \eta_{s}\left(z_{s}|v^{t-1}\right), & t\geq 0\wedge |s|=1,\\ \frac{\eta_{s}\left(z_{s}|v^{t-1}\right)f\left(v_{t}|v^{t-1},s,z_{s}\right)\prod_{s^{\prime }\in \mathrm{Ch}\left(s\right)}q{\left(v_{t}|v^{t-1},s^{\prime }\right)}^{z_{ss^{\prime }}}}{q(v_{t}|v^{t-1},s)}, & \mathrm{otherwise}. \end{array}\right. \end{array}$$

By substituting the above general updating formula,

$$\begin{array}{cc} \prod_{s\in S^{m}}\eta_{s}\left(z_{s}|v^{t}\right) & =\prod_{s\in I^{m}}\frac{\eta_{s}\left(z_{s}|v^{t-1}\right)f\left(v_{t}|v^{t-1},s,z_{s}\right)\prod_{s^{\prime }\in \mathrm{Ch}\left(s\right)}q{\left(v_{t}|v^{t-1},s^{\prime }\right)}^{z_{ss^{\prime }}}}{q(v_{t}|v^{t-1},s)} \end{array}$$

(A15) $$\begin{array}{cc} \; & \;\times \prod_{s\in L^{m}}\frac{\eta_{s}\left(z_{s}|v^{t-1}\right)f\left(v_{t}|v^{t-1},s,z_{s}\right)\prod_{s^{\prime }\in \mathrm{Ch}\left(s\right)}q{\left(v_{t}|v^{t-1},s^{\prime }\right)}^{0}}{q(v_{t}|v^{t-1},s)} \end{array}$$

(A16) $$\begin{array}{cc} \; & =\frac{1}{q(v_{t}|v^{t-1},s_{\lambda })}\prod_{s\in S^{m}}\eta_{s}\left(z_{s}|v^{t-1}\right)\prod_{s\in S^{m}}f\left(v_{t}|v^{t-1},s,z_{s}\right) \end{array}$$

(A17) $$\begin{array}{cc} \; & =\frac{p\left(m|v^{t-1}\right)p\left(v_{t}|v^{t-1},m\right)}{p(v_{t}|v^{t-1})}=p\left(m|v^{t}\right) \end{array}$$

In the above operation, (A15) was a telescoping product, i.e., $q(v_{t}|v^{t-1},s)$ appeared at once in each of the denominator and the numerator. Therefore, we canceled them except for $q(v_{t}|v^{t-1},s_{\lambda })$. (A16) is because of (A8), (A10) and (A11), where (A8) and (A11) are the induction hypotheses. □

## References

[1] Nakahara Y., Matsushima T. (2021). A Stochastic Model for Block Segmentation of Images Based on the Quadtree and the Bayes Code for It. Entropy.

[2] Weinberger M.J., Seroussi G., Sapiro G. (2000). The LOCO-I lossless image compression algorithm: Principles and standardization into JPEG-LS. IEEE Trans. Image Process..

[3] Wu X., Memon N. (1997). Context-based, adaptive, lossless image coding. IEEE Trans. Commun..

[4] Matsuda I., Ozaki N., Umezu Y., Itoh S. Lossless coding using variable block-size adaptive prediction optimized for each image. Proceedings of the 2005 13th European Signal Processing Conference.

[5] Huffman D.A. (1952). A Method for the Construction of Minimum-Redundancy Codes. Proc. IRE.

[6] Rissanen J., Langdon G. (1981). Universal modeling and coding. IEEE Trans. Inf. Theory.

[7] Nakahara Y., Matsushima T. Autoregressive Image Generative Models with Normal and t-distributed Noise and the Bayes Codes for Them. Proceedings of the 2020 International Symposium on Information Theory and Its Applications (ISITA).

[8] Nakahara Y., Matsushima T. Hyperparameter Learning of Stochastic Image Generative Models with Bayesian Hierarchical Modeling and Its Effect on Lossless Image Coding. Proceedings of the 2021 IEEE Information Theory Workshop (ITW).

[9] Nakahara Y., Matsushima T. (2020). Bayes code for two-dimensional auto-regressive hidden Markov model and its application to lossless image compression. Proceedings of the International Workshop on Advanced Imaging Technology (IWAIT) 2020.

[10] Berger J.O. (2013). Statistical Decision Theory and Bayesian Analysis.

[11] Matsushima T., Inazumi H., Hirasawa S. (1991). A class of distortionless codes designed by Bayes decision theory. IEEE Trans. Inf. Theory.

[12] Clarke B.S., Barron A.R. (1990). Information-theoretic asymptotics of Bayes methods. IEEE Trans. Inf. Theory.

[13] Matsushima T., Hirasawa S. Reducing the space complexity of a Bayes coding algorithm using an expanded context tree. Proceedings of the 2009 IEEE International Symposium on Information Theory.

[14] Sullivan G.J., Ohm J., Han W., Wiegand T. (2012). Overview of the High Efficiency Video Coding (HEVC) Standard. IEEE Trans. Circuits Syst. Video Technol..

[15] Nakahara Y., Saito S., Kamatsuka A., Matsushima T. Probability Distribution on Rooted Trees. Proceedings of the 2022 IEEE International Symposium on Information Theory.

[16] Martín G. Range encoding: An algorithm for removing redundancy from a digitised message. Proceedings of the Video and Data Recording Conference.

[17] Kuhn M. JBIG-KIT. https://www.cl.cam.ac.uk/~mgk25/jbigkit/.

[18] Langley A. jbig2enc. https://github.com/agl/jbig2enc.

[19] Image Repository of the University of Waterloo. http://links.uwaterloo.ca/Repository.html.

[20] Skodras A., Christopoulos C., Ebrahimi T. (2001). The JPEG 2000 still image compression standard. IEEE Signal Process. Mag..

[21] Weinlich A., Amon P., Hutter A., Kaup A. (2016). Probability Distribution Estimation for Autoregressive Pixel-Predictive Image Coding. IEEE Trans. Image Process..

